# A Comparative Study of Sodium Alginate and Plasma Thrombin Cell Block in Diagnostic Cytopathology

**DOI:** 10.7759/cureus.70158

**Published:** 2024-09-25

**Authors:** Kanika Sachar, Vijayalaxmi Patil

**Affiliations:** 1 Pathology, Shri B.M. Patil Medical College, Hospital & Research Centre, BLDE (Deemed to be University), Vijayapura, IND

**Keywords:** cell blocks, fine needle aspiration cytology, immunohistochemistry, plasma thrombin, serous fluid, sodium alginate

## Abstract

*Background*: Cell block is an indispensable supplement in the practice of cytopathology. The diagnostic utility of cytology specimens is significantly impacted by the capacity to generate sufficient cell blocks obtained from concentrated fluid samples or fine-needle aspiration specimens after routine processing. This routine processing involves getting directed passes to produce a cell block, especially if the cytopathologist believes additional immunocytochemical stains and/or molecular studies would be required.

*Objective*: This study compared two methods of cell block preparation: the sodium alginate (SA) method and the plasma thrombin (PT) method. A comparison was made regarding overall cellularity, morphological preservation, and concealed artifacts.

*Methodology*: This cross-sectional study evaluated 104 serous fluid samples and fine-needle aspirates. Cell blocks were prepared for each sample using the plasma thrombin and sodium alginate technique. The formalin-fixed, paraffin-embedded cell blocks were subjected to histochemical staining with hematoxylin and eosin, and slides were assessed for cellularity, artifacts, and morphological preservation.

*Results*: The study utilized chi-square tests to analyze cellularity, morphology, and artifact presence, demonstrating significant differences in cellularity and artifacts between the two methods, with the sodium alginate method showing more cellularity and more artifacts, while morphologically, there was no significant difference between the two methods.

*Conclusion*: Our study’s findings have practical implications for cytopathologists. We conclude that, compared to plasma thrombin methodology, the sodium alginate cell block technique yields higher cellularity, while there was no difference in morphology. Even though artifacts were more prevalent in sodium alginate cell blocks than in plasma thrombin cell blocks, our study suggests that the former can be a better alternative for cell block examination.

## Introduction

Fine-needle aspiration cytology (FNAC) is a convenient, affordable, and safe modality for tissue diagnosis of subcutaneous and other tumors. It is a treasured tool providing an effective, prompt, and accurate preoperative assessment of the lesions of the breast, skin, soft tissue, thyroid, lungs, lymph nodes, and liver [1]. This cytodiagnostic technique is more beneficial and considered more specific and sensitive than surgical biopsies when limited resources are available [2].

More specific diagnoses can be obtained on fine-needle aspirates (FNAs) by adding immunohistochemistry and other molecular techniques to the prepared cell block specimens [2]. Exfoliative specimens such as effusions, bronchial washings, and FNAs, particularly from deep-seated lesions, are commonly used to prepare cell blocks. Cell blocks are a crucial component of cytology. Still, their value is perhaps more widely understood now than ever because of their important function in ancillary testing, especially molecular diagnostics [3].

Being one of the earliest techniques, the cell block technique has undergone numerous variations. Still, the method hasn't garnered much attention because there isn't a defined procedure. Despite the documented benefits of enhanced cellular morphology through histology techniques and the concentration of minimal amounts of cellular material in a compact region that can be analyzed quickly, there is a lack of comparative studies that rigorously evaluate the efficacy of sodium alginate versus plasma thrombin in cell block preparation [4].

Cell blocks are becoming increasingly significant as substrates for prognostic and predictive marker testing in the era of customized treatment. The value of cytologic sampling goes beyond diagnostic evaluation because it can eliminate the need for further steps to obtain tissue for ancillary tests or because it might be the sole material available for such testing [5]. Almost any cytology sample can be used to create cell blocks, including scraps from traditional smears or leftover material from liquid-based preparations [6].

Over the years, numerous cell block approaches, including manual and automated, have been created and reviewed, each with its own unique scope, fixatives, processing, and embedding methods [1,7]. Few research studies contrast the enzyme-based plasma thrombin cell block method with chemical-based sodium alginate approaches, even though numerous studies have examined the various methods of cell block preparation in the literature [7]. The present study compared the cell blocks prepared by the plasma thrombin and sodium alginate methods regarding cellularity, morphology, and the presence of artifacts.

## Materials and methods

This was a hospital-based cross-sectional study that compared two different methods of cell block preparation. A total of 104 samples, including fine-needle aspirates and serous fluids, were included in the study, conducted from September 2022 to May 31, 2024, in the cytopathology section of the Pathology Department of the Shri B. M. Patil Medical College, Hospital and Research Centre, which is part of the BLDE (Deemed to be University), Vijayapura.

The study aimed to prepare cell blocks using the plasma thrombin and sodium alginate methods to compare the two methods for various cytological features such as cellularity, cytomorphological preservation, and presence of artifacts. Fine-needle aspirate samples of patients and body fluids received in the cytology section were included in the study, while inadequate aspirates and insufficient fluid samples were excluded. The institutional ethical clearance was obtained, and the approval number is BLDE(DU)/IEC/682/2022-2023.

Cell sediment was prepared from the serous fluids by centrifuging the fluid for 10 minutes at 1500 rpm. Cell sediment of fine-needle aspirates was obtained by rinsing the needle with normal saline and centrifuging the fluid.

A 5 ml of sediment was taken in a test tube to prepare the cell block using the plasma thrombin method, to which 100 μl of normal human plasma was added, followed by 50μl of thrombin. After gently removing the clot from the tube, it was processed further as a small biopsy to obtain a paraffin-embedded cell block.

To prepare the cell block using the sodium alginate method, 3 ml of 10% neutral buffered formalin and 0.5 ml of 1% sodium alginate were added to the cell sediment. This step was followed by centrifugation for 5 min at 3000 rpm and adding 0.5 ml 1 M calcium chloride. In approximately 5-10 min, the gel-like clot was formed, which was processed as a small biopsy to obtain a paraffin-embedded cell block.

Sections from paraffin-embedded plasma thrombin and sodium alginate cell blocks were prepared, stained with hematoxylin and eosin and examined for cellularity, morphology, and artifacts. The cellularity was assessed as 1+, 2+, and 3+ cellularity, with 3+ cellularity being awarded for many macro-fragments or numerous large aggregates of cells and dispersed cells; 2+ cellularity for occasional or few micro-fragments and few aggregates of cells and dispersed cells and 1+ cellularity for only dispersed cells with no fragments or large aggregates.

The cell was assessed for morphology to determine if the morphology was optimal or suboptimal. Optimal morphology included proper nuclear and cytoplasmic staining. The presence of artifacts, if any, was also recorded in the cell blocks prepared by the plasma thrombin and sodium alginate method.

## Results

**Figure 1:**
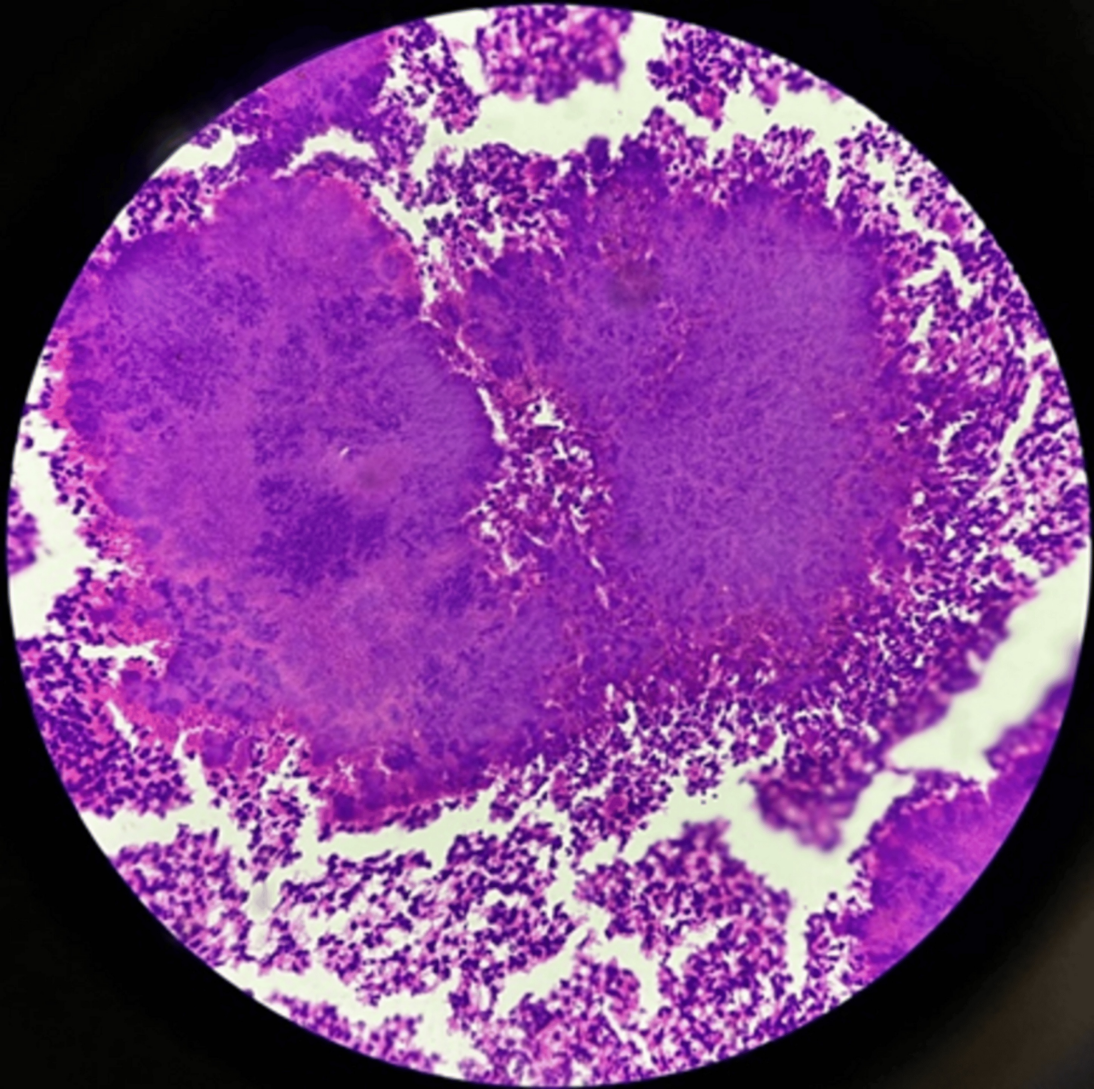
Sodium alginate cell block of submental swelling aspirate demonstrating actinomycetes colonies with surrounding neutrophils, indicating effective cytomorphological preservation and 3+ cellularity (hematoxylin and eosin stain, 40X).

**Figure 2:**
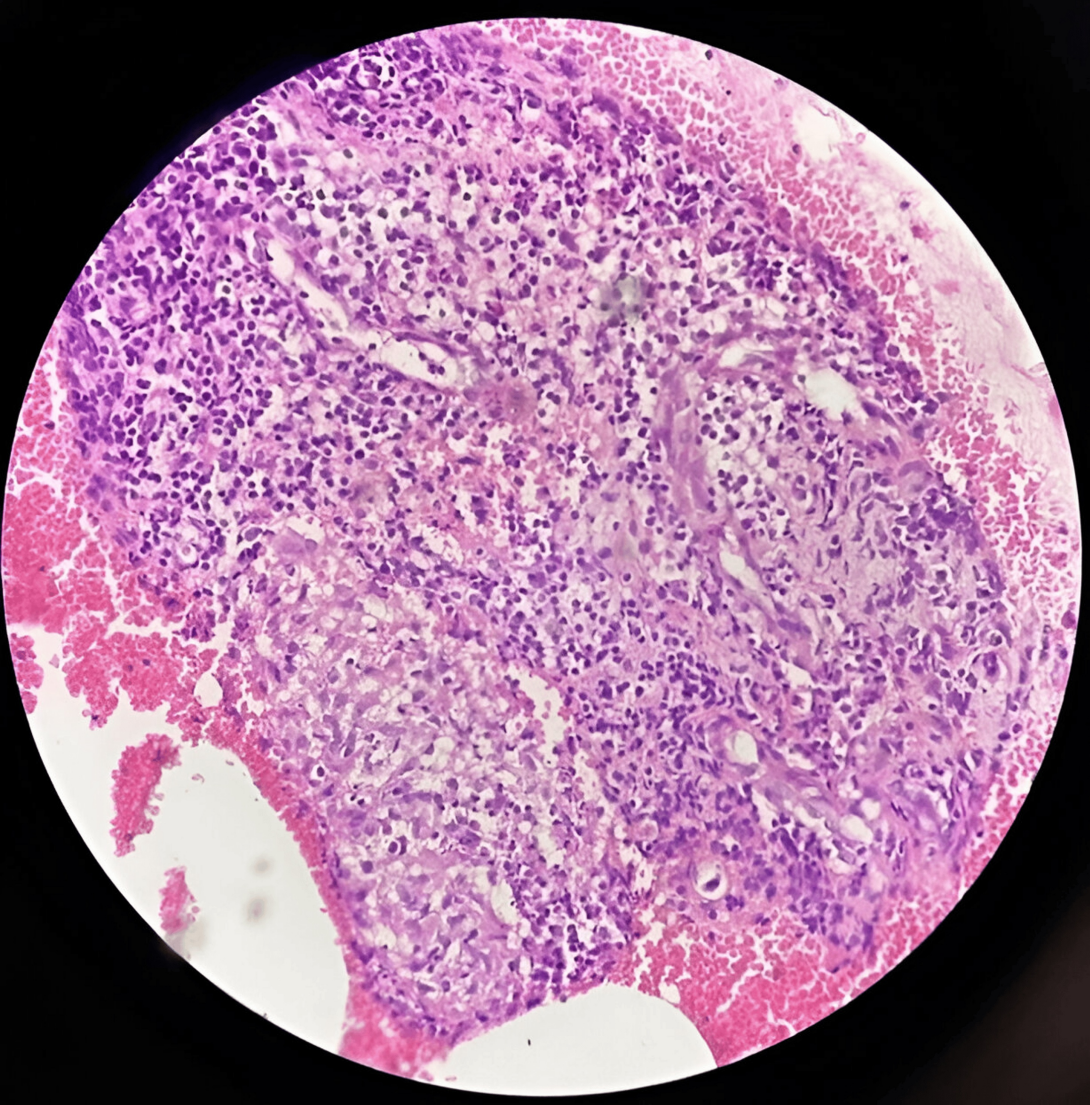
Sodium alginate cell block of breast aspirate demonstrating granulomas with effective cytomorphological preservation, having 3+ cellularity (hematoxylin and eosin stain, 40X).

**Figure 3:**
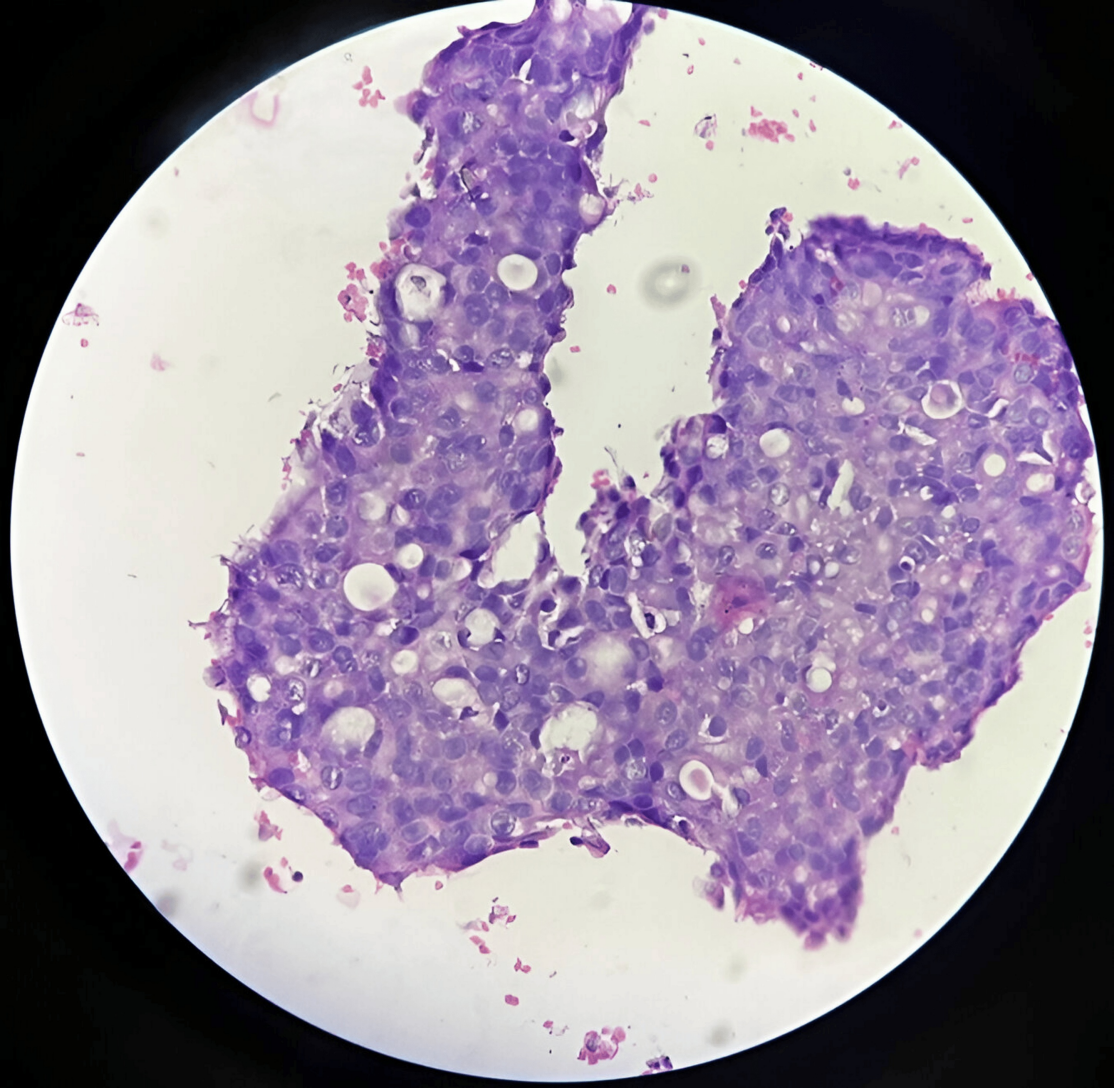
Plasma thrombin cell block of salivary gland aspirate demonstrating effective cytomorphological preservation and 2+ cellularity (hematoxylin and eosin stain,, 40X).

**Figure 4:**
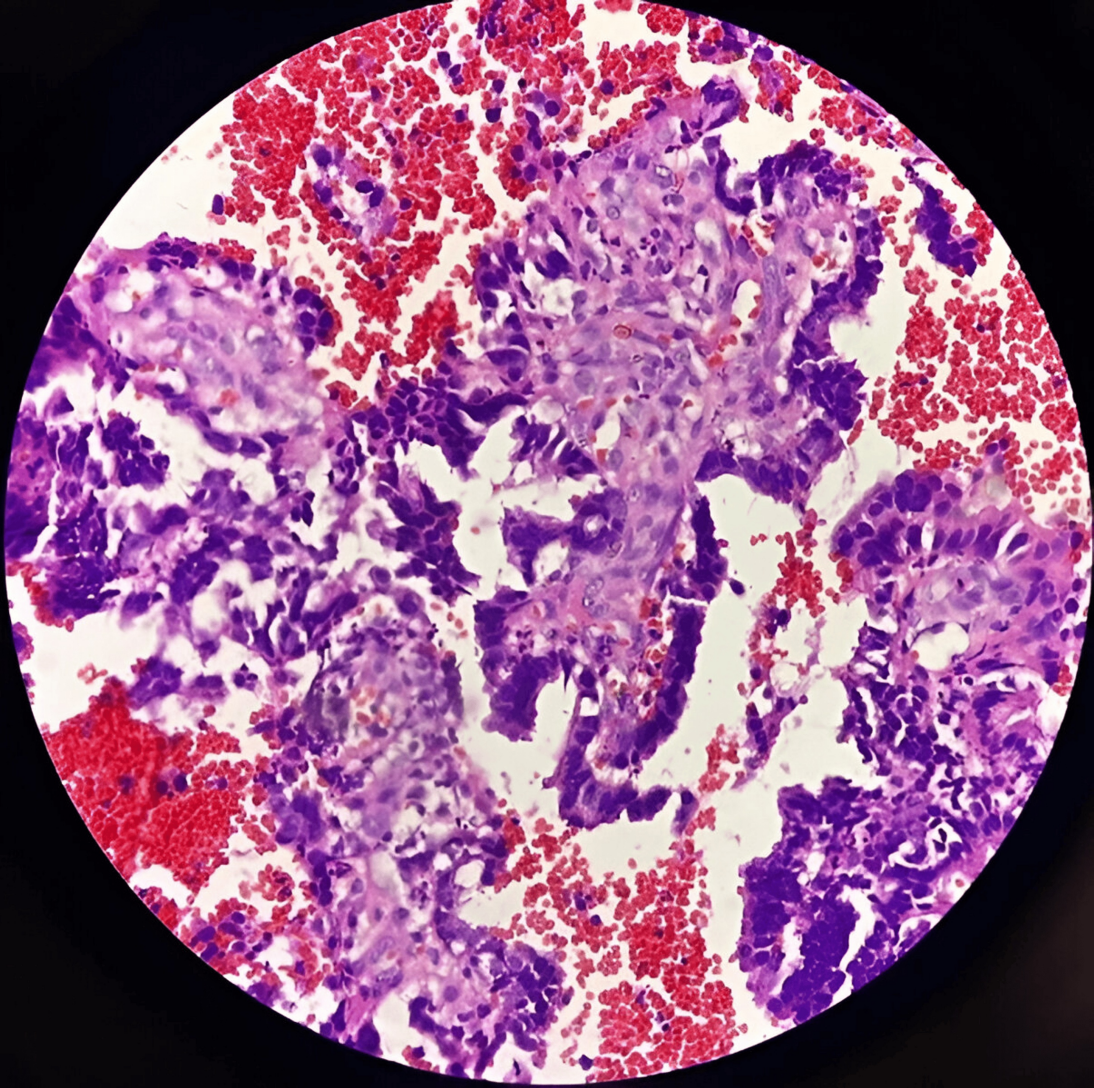
Plasma thrombin cell block of lymph node aspirate demonstrating papillae, indicating effective cytomorphological preservation and 3+ cellularity (hematoxylin and eosin stain, 40X).

Cell blocks were prepared from 104 samples using plasma thrombin and sodium alginate methods and were subsequently examined. The sample set included fine-needle aspirates and serous fluids. Table 1 compares the cellularity between cell blocks prepared by the plasma thrombin and sodium alginate methods. Of these 104 samples, 77 (74%) were serous fluids, and 27 (25.9%) were fine-needle aspirates. Among the 104 samples, 59 (56%) cell blocks exhibited 1+ cellularity with the plasma thrombin method. Within these, 35 out of 47 cell blocks (74.5%) showed 1+ cellularity, 20 out of 40 cell blocks (50%) exhibited 2+ cellularity, and four out of 17 cell blocks (23.5%) demonstrated 3+ cellularity when using the sodium alginate method as shown in Figures 1, 2. Additionally, 33 (31.7%) cell blocks showed 2+ cellularity with the plasma thrombin method illustrated in Figure 3. Within this group, five out of 17 cell blocks (29.4%) showed 3+ cellularity, 18 out of 40 cell blocks (45%) showed 2+ cellularity, and 10 out of 47 cell blocks (21.3%) showed 1+ cellularity with the sodium alginate method. Finally, 12 out of 104 cell blocks (11.5%) showed 3+ cellularity using the plasma thrombin method (see Figure 4). Of these, eight out of 17 cell blocks (47%) also demonstrated 3+ cellularity with the sodium alginate method. In comparison, two out of 40 cell blocks (5%) showed 2+ cellularity and two out of 47 (4.3%) showed 1+ cellularity using the sodium alginate method. A chi-square test was selected for its suitability in comparing categorical data, yielding a significant p-value (0.001), thereby confirming that the sodium alginate method provides superior cellularity.

**Table 1:** Comparison of the cellularity between cell blocks prepared by the plasma thrombin and sodium alginate methods. * Statistically significant (p<0.05)

Plasma thrombin cellularity	Sodium alginate cellularity				Chi-square	P value
	1+	2+	3+	Total		
1+	35	20	4	59	32.328	0.001*
	74.5%	50.0%	23.5%	56.7%		
2+	10	18	5	33		
	21.3%	45.0%	29.4%	31.7%		
3+	2	2	8	12		
	4.3%	5.0%	47.1%	11.5%		
Total	47	40	17	104		
	100.0%	100.0%	100.0%	100.0%		

Table 2 shows the detailed comparison of cellular morphology between cell blocks prepared by the plasma thrombin and sodium alginate methods. Among the 104 samples analyzed, 102 (98%) cell blocks exhibited optimal morphology and two (1.9%) cell blocks showed suboptimal morphology using the plasma thrombin method. In comparison, 100 out of 104 cell blocks (96%) prepared with the sodium alginate method were of optimal morphology, while four out of 104 cell blocks (3.8%) were suboptimal. A chi-square test was selected for its suitability in comparing categorical data, yielding no statistically significant p-value (0.775), thereby confirming that there is no morphological difference seen with cell blocks prepared by the plasma thrombin and sodium alginate methods.

**Table 2:** Comparison of cellular morphology between cell blocks prepared by plasma thrombin method and sodium alginate method.

Plasma thrombin morphology	Sodium alginate morphology			Chi-square	P value
	Optimal	Suboptimal	Total		
Optimal	98	4	102	.082	.775
	98.0%	100.0%	98.1%		
Suboptimal	2	0	2		
	2.0%	0.0%	1.9%		
Total	100	4	104		
	100.0%	100.0%	100.0%		

Table 3 compares artifacts between cell blocks prepared by plasma thrombin and sodium alginate methods. None of the 58 cell blocks (55.8%) prepared using the plasma thrombin method showed artifacts. Of these, 41 out of 51 cell blocks (80.4%) prepared with the sodium alginate method displayed no artifacts, while 17 out of 53 (32.1%) showed artifacts. Conversely, 46 cell blocks (44.2%) prepared with the plasma thrombin method did have artifacts; of these, 36 out of 53 cell blocks (67.9%) showed artifacts when evaluated with the sodium alginate method, while 10 out of 51 cell blocks (19.6%) showed no artifacts. A chi-square test was selected for its suitability in comparing categorical data, yielding a significant p-value (0.001), thereby confirming that the sodium alginate method provides superior cellularity.

**Table 3:** Comparison of artifacts between cell blocks prepared by plasma thrombin and sodium alginate methods. * Statistically significant (p<0.05)

Plasma thrombin artifacts	Sodium alginate artifacts			Chi-square	P value
	Absent	Present	Total		
Absent	41	17	58	24.597	.0001*
	80.4%	32.1%	55.8%		
Present	10	36	46		
	19.6%	67.9%	44.2%		
Total	51	53	104		
	100.0%	100.0%	100.0%		

As shown in Figure 1, the sodium alginate cell block of submental swelling aspirate demonstrates actinomycetes colonies with surrounding neutrophils, indicating effective cytomorphological preservation and 3+ cellularity. Figure 2 illustrates a sodium alginate cell block of breast aspirate, demonstrating granulomas with effective cytomorphological preservation and 3+ cellularity. As depicted in Figure 3, the plasma thrombin cell block of salivary gland aspirate shows effective cytomorphological preservation and 2+ cellularity. Figure 4 demonstrates the plasma thrombin cell block of lymph node aspirate, indicating papillae with effective cytomorphological preservation and 3+ cellularity.

## Discussion

Pathology in the current times strives to provide the most thorough diagnosis using the least invasive techniques. These initiatives have allowed for the diagnosis of patients using progressively smaller tissue samples. The pursuit of non-invasive diagnosis led to a focus on cytological examinations. However, cytological examination, particularly fine-needle aspiration cytology, might not be enough to provide a diagnosis, and there may be a chance of receiving an intermediate or false-negative result. Research has demonstrated that even with meticulous preparation, cytological analysis of specimens using smears leaves behind a significant residue that is not examined further but may contain important diagnostic information [6,8].

Cytopreparatory approaches have been created to make better use of these cytological materials. The most recent of these methods is acquired by centrifuging cytological material, embedding it in paraffin and then studying it [8].

Several cell block preparation methods use different media to transport, rinse, and fix specimens. The fixative of choice for most people is neutral buffered formalin. There are several ways to prepare cell blocks; plasma thrombin is the most widely used method. By raising the specimen's diagnostic yield and lowering the need for repeated fine-needle aspiration treatments, cell block material aids in diagnosis [9].

Nonetheless, the importance of cell blocks keeps expanding as new immunohistochemical (IHC) markers and technological developments like multicolor IHC and the subtractive coordinate immunoreactivity pattern approach are created [10]. They provide a readily accessible tissue source for molecular testing, which is becoming a more common component of cancer treatment [4].

An ideal cell block should be able to preserve the architecture and cytomorphology while consolidating the finest needle aspirate material possible, including micro-fragments and single-distributed cells. As previously indicated, several studies have examined the various approaches to manufacturing cell blocks; however, no study has contrasted the chemically based sodium alginate cell block method with the enzyme-based plasma thrombin cell block method [7].

In our study, we effectively generated cell blocks that demonstrated excellent cytomorphologic preservation by using sodium alginate, as recommended by Sano et al. [11]. A few writers have also effectively produced sodium alginate cell blocks in the past, demonstrating excellent cellular features and well-preserved architecture. Nevertheless, no comparison has been made with the more widely used plasma thrombin method of preparing cell blocks [11-13].

This study, comprising 104 samples, underscores the potential of sodium alginate as a superior alternative in cell block preparation. However, further research is warranted to explore the applicability of these findings across diverse cytology samples and in a clinical setting. We studied 77 (74%) samples of serous fluids and 27 (25%) samples of fine-needle aspirates. Of those 77 samples, 42 (54%) samples were of pleural fluid, 30 (38%) samples were of ascitic fluid, and five (6%) samples were of bronchoalveolar lavage. In our study, cell blocks were prepared using plasma thrombin and sodium alginate methods. Later, the histologic slides prepared from cell blocks were evaluated for cellularity, morphology, and presence of artifacts. Despite the documented benefits of enhanced cellular morphology through histology techniques, there is a lack of comparative studies that rigorously evaluate the efficacy of sodium alginate versus plasma thrombin in cell block preparation.

Comparable cell recovery was seen, with 11.5% of plasma thrombin cell blocks and 16.3% of sodium alginate cell blocks exhibiting 3+ cellularity. The cytomorphology of the sections stained with hematoxylin and eosin from the two cell block procedures - the plasma thrombin and sodium alginate methods - showed good cellular preservation of 98.1% and 96.2%, respectively. Of the sections, 51% of the sodium alginate cell blocks had pale pink artifacts, but only 44.2% of plasma thrombin cell blocks had them. This difference was statistically significant. Except for these artifacts, the cell blocks prepared by the sodium alginate method were identical to the plasma thrombin approach in terms of morphology and architecture.

Gupta et al. studied 80 cases of 45 fine-needle aspiration cytologies and 35 serous effusions. Cell blocks were prepared using the plasma thrombin technique from 21 fine-needle aspiration cytology specimens and 19 effusion cytology specimens. In comparison, 24 fine-needle aspirates and 16 effusion cytology specimens were studied using sodium alginate. The hematoxylin and eosin-stained sections from the formalin-fixed paraffin-embedded cell blocks from both methods were evaluated for cellularity, artifacts, and morphological preservation. They concluded that sodium alginate cell blocks could be an excellent alternative for plasma thrombin cell blocks in routine practice with no quality compromise on cellularity and morphological preservation [7].

Khan et al. compared the cell blocks and fine-needle aspirate samples to evaluate cytomorphology and immunocytochemistry (ICC) stains and determine the effectiveness of the cell block. They found that fine-needle aspiration smears had better nuclear and morphologic characteristics than the cell block, while cell block samples had better ICC staining due to lack of background and aberrant staining. They concluded that cell blocks and fine-needle aspirate smears support each other, and both are indicated in the diagnostic work-up of patients [14].

Sarkar et al. evaluated the validity of a combined approach of routine cytological staining methods, cell block techniques, and immunohistochemistry on cell block sections in differentiating adenocarcinoma cells from reactive mesothelial cells for histology. To increase the diagnostic accuracy of suspicious or malignant ascites cases as much as possible, they advised combining cytology on standard smears with cell block and IHC [15]. However, IHC on cell blocks was not used in our study, which is a limitation.

## Conclusions

In the present study, we evaluated cell blocks of fine-needle aspirates and serous fluid samples prepared using sodium alginate and plasma thrombin. The cell blocks were assessed for cellularity, morphology, and presence of artifacts. We found that cellular yield was higher for cell blocks prepared by the sodium alginate method compared to the plasma thrombin method. At the same time, sodium alginate cell blocks also had more artifacts than plasma thrombin cell blocks. Morphologically, no significant difference was noted between the cell blocks prepared with sodium alginate and plasma thrombin. Hence, we conclude that the sodium alginate method offers a better alternative for preparing the cell blocks.
